# Interaction Between Immigration, Physical Activity, Mental Health, and All-Cause Mortality Among US Adults

**DOI:** 10.1001/jamanetworkopen.2025.36371

**Published:** 2025-10-08

**Authors:** David Adzrago, Timothy S. McNeel, Typhanye V. Dyer, Yvonne Commodore-Mensah, Maryam Elhabashy, Cameron K. Ormiston, Benedicta Osafo-Darko, Donna J. Cherry, J. Michael Wilkerson, Faustine Williams

**Affiliations:** 1 Division of Intramural Research, National Institute on Minority Health and Health Disparities, National Institutes of Health, Bethesda, Maryland; 2 Information Management Services, Inc, Calverton, Maryland; 3 Department of Epidemiology and Biostatistics, University of Maryland at College Park, College Park; 4 School of Nursing, Johns Hopkins University, Baltimore, Maryland; 5 Department of Medical Education, Icahn School of Medicine at Mount Sinai, New York City, New York; 6 Department of Behavioral and Community Health, University of Maryland at College Park, College Park; 7 Department of Social Work, East Tennessee State University, Johnson City; 8 Department of Health Promotion and Behavioral Sciences, The University of Texas Health Science Center at Houston, Houston

## Abstract

**Importance:**

Immigration, physical activity, and mental health have been reported to affect mortality risk. Nonimmigrants, people who lack physical activity, and those with mental health conditions are at a higher mortality risk. However, the joint association of immigration, physical activity, and mental health with mortality risk is unclear.

**Objective:**

To estimate the interaction among immigration status, physical activity status, and serious psychological distress (SPD) and its association with all-cause mortality risk among US adults during a 22-year follow-up.

**Design, Setting, and Participants:**

In this national cohort study, public-use data from the 1998-2018 National Health Interview Surveys were linked to the prospective 2018-2019 National Death Index data. This population-based study included a nationally representative sample of civilian noninstitutionalized US adults 18 years or older. Data were collected from January 1, 1998, to December 31, 2019, and analyzed from August 10, 2023, to July 15, 2025.

**Exposures:**

Immigration status (immigrant or nonimmigrant), physical activity (inactive or insufficiently active or physically active), and SPD (determined using the 6-item Kessler Psychological Distress Scale, with a total score of ≥13 indicating SPD).

**Main Outcomes and Measures:**

All-cause mortality status (deceased or alive) constituted the primary outcome. Cox proportional hazards regression models were used to examine mortality risk associated with the exposures.

**Results:**

A total of 587 931 adults were included in the analysis. Weighted proportions included 37.2% (95% CI, 37.0%-37.4%) aged 35 to 54 years, 51.9% (95% CI, 51.8%-52.1%) female, 83.8% (95% CI, 83.5%-84.1%) nonimmigrant, 53.7% (95% CI, 53.4%-54.0%) with insufficient physical activity, and 96.8% (95% CI, 96.7%-96.8%) with no SPD. The Cox proportional hazards regression analysis showed a significant 3-way interaction among immigration status, physical activity, and SPD (Wald *F*_4_ = 14.12; *P* < .001). There was a lower expected mortality risk among immigrants with SPD who engaged in physical activity (HR, 6.08; 95% CI, 3.97-8.19) compared with immigrants with SPD who lacked physical activity (HR, 10.50; 95% CI, 8.93-12.08) as well as nonimmigrants who lacked physical activity regardless of SPD status (No SPD HR, 13.03; 95% CI, 12.16-13.90 and SPD HR, 14.13; 95% CI, 13.04-15.21) or who engaged in physical activity with SPD (HR, 11.44; 95% CI, 9.89-12.99). There were no significant differences between immigrants without SPD who engaged in physical activity or immigrants without SPD who lacked physical activity and nonimmigrants who engaged in physical activity without SPD.

**Conclusions and Relevance:**

In this population-based cohort study, the results showed a joint association of immigration status, physical activity, and SPD with all-cause mortality. Immigrants and especially nonimmigrants might benefit from tailored behavioral health interventions aimed at increasing physical activity and reducing SPD to mitigate disparities in mortality risk.

## Key Points

**Question:**

Are interactions between immigrant status, physical activity, and serious psychological distress (SPD) associated with all-cause mortality?

**Findings:**

This cohort study of 587 931 US adults from 1998 to 2019 found a significant interaction among immigration, physical activity, and SPD status. Immigrants with SPD who engaged in physical activity had a lower all-cause mortality risk compared with immigrants with SPD who lacked physical activity, as well as nonimmigrants who lacked physical activity regardless of SPD status or nonimmigrants who engaged in physical activity with SPD.

**Meaning:**

These findings suggest that interventions considering interactions among immigration, physical activity, and SPD status may be factors associated with reducing mortality risks.

## Teaser

This cohort study examines the risks of mortality associated with serious psychological distress, physical activity, and immigration status among the US population.

## Introduction

Lack of physical activity, including leisure time and nonleisure time, has been associated with chronic conditions, poor mental health, behavioral health problems, and premature mortality.^1,2,3,4,5,6,7^ However, the joint effects of physical activity and mental health on mortality risk are poorly understood, especially among immigrants, a growing population that remains largely understudied. Physical activity has been associated with improved mental health, including decreased risks of anxiety, depression, and psychological distress, as well as reduced risks of behavioral health problems (eg, substance use).^4,5,6^ Individuals with negative mental health outcomes, such as anxiety and depression, or psychological distress have elevated risks of years of life lost and mortality.^8,9^ Mental health problems also impose significant economic burdens, with total projected mental health spending in the US increasing significantly from $147.4 billion in 2009 to $238.4 billion in 2020—a 38.2% increase, equating to a 4.5% average annual growth rate.^10^ Moreover, research indicates that immigration significantly contributes to disparities in US mortality rates.^11^ Since 1990, the immigrant population has experienced lower mortality rates and higher life expectancy than the US-born population.^11^ The literature reports that the immigrant population exhibits better physical and mental health outcomes than the US-born population, despite the immigrant population experiencing higher socioeconomic disadvantages (eg, unemployment, language barriers, discrimination).^12,13^ Socioeconomic deprivation increases the risk of mental health disorders and reduces physical activity levels.^1,9,14,15^ Consequently, studies state that immigrants engage in less physical activity than US-born individuals.^16,17,18,19^ In particular, studies indicate that immigrants engage in more transportation and occupational physical activities, but participate less frequently in leisure-time physical activity.^20,21,22,23^ Thus, it is critical to consider how physical activity, mental health, and immigration are jointly associated with mortality risks among the US population. In this study, we examine the risks of mortality associated with serious psychological distress (SPD), physical activity, and immigration status among the US population. Specifically, we examine the interactions between (1) nativity status (hereafter, immigration status) and physical activity, (2) immigration status and SPD, (3) physical activity and SPD, and (4) immigration status, physical activity, and SPD.

## Methods

### Data Source, Study Design, and Study Sample

In this cohort study, we followed the Strengthening the Reporting of Observational Studies in Epidemiology (STROBE) reporting guidelines. This study used publicly available deidentified secondary data; thus, no institutional review board approval or informed consent was needed in accordance with Common Rule 45 CFR §46.

We analyzed public-use data from the 1998-2018 National Health Interview Surveys (NHIS) linked to the 2018-2019 death certificate data from the National Death Index (NDI).^11^ Data analysis was conducted from August 10, 2023, to July 15, 2025. The NHIS is a household interview survey that targets the civilian noninstitutionalized US population residing within the 50 states and Washington, DC, at the time of the interview.^24,25,26^ The NHIS collects health information, including mental health, substance use (eg, alcohol, tobacco), and sociodemographic characteristics.^24,25,26^ The sampling methodology involves a stratified multistage sample, with counties or adjacent counties as the primary sampling units, clusters of houses as the secondary sampling units, and households and randomly selected individuals within households as the final or tertiary sampling units.^24,26^ The National Center for Health Statistics (NCHS) established the NDI, a central computerized database containing death record information in the State vital statistics offices, as a resource for health and medical researchers to assess their study participants’ mortality records.^25^ The NCHS has linked the NHIS participants’ records with the NDI’s records, using the participants’ identifiers such as Social Security number, first name, middle initial, last name, father’s surname, birthdate, sex, race and ethnicity, state or country of birth, and state of residence.^25^ Race and ethnicity were included in the analysis because of the known disparities in life expectancy and mortality risks. The NHIS-NDI data were matched (using deterministic and probabilistic approaches) based on the participants’ identifiers.^25,27^ The NCHS developed the public-use versions of the linked data to protect the confidentiality of the participants and increase data access. The public-use data provide mortality follow-up information from the date of survey through December 31, 2019.^24,25^ The Integrated Public Use Microdata Series NHIS database was used to combine and harmonize the data across survey years.^24^

Data for this study were collected from January 1, 1998, to December 31, 2019. These analyses were restricted to adult samples who provided sufficient data for linkage to the NDI (N = 611 505). Of the total sample of adults who provided sufficient data, 23 574 (3.9%) had unknown values or responses on the key baseline variables (ie, psychological distress, immigration status, and physical activity) used in the study. The analytical sample (n = 587 931) excluded respondents who had missing values for the key baseline variables.

### Measures

#### Outcome

Mortality status (ie, all-cause mortality status) was determined based on the NDI records. Each survey participant who is linkage-eligible for mortality follow-up is assigned a vital status (deceased or alive). The semiclosed failure time interval started on the date of the interview and ended on the date of death or December 31, 2019, for participants who were alive.

#### Exposures

For immigration status, the participants were categorized as immigrants if they were born outside the US or in US territories. Those born in one of the 50 US states or Washington, DC, were categorized as nonimmigrants. Serious psychological distress (SPD) status was assessed using the Kessler Psychological Distress Scale, a 6-item scale with total scores ranging from 0 to 24.^28,29^ The extant literature reports a score of 13 or more on the Kessler Psychological Distress Scale as an indicator of diagnosable mental illness’ presence (ie, SPD), with increased risks of disability and mortality.^28,29,30^ The scale assesses how often the participants feel sad, nervous, restless, hopeless, worthless, or that everything was an effort in the past 30 days.^28,29^ Leisure-time physical activity was considered sufficient if the participants met the goal of 150 minutes per week of moderate activity, 75 minutes per week of vigorous activity, or an equivalent combination of the two. Physical activity status was created according to the weekly physical activity duration recommended in the 2018 Health and Human Services Physical Activity Guidelines.^31^

#### Covariates

Based on previous studies,^1,11,14,25,32^ several self-reported sociodemographic characteristics (age, sex, race and ethnicity, educational attainment, employment status, income-to-poverty ratio, health insurance status, region of residence, and body mass index) and behavioral health (smoking and alcohol consumption or substance use) were included as covariates.

### Statistical Analysis

We computed descriptive statistics to describe the proportion of the participants and estimate the mortality rates by the exposure variables and the covariates. To illustrate the survival probability at a certain time interval during the follow-up, Kaplan-Meier survival curves were estimated for combinations of immigration SPD, and physical activity status. We also used the Cox proportional hazards regression to assess 6 survival models. Model 1 evaluated the survival (in this case, death) by immigration, physical activity, and SPD status, without adjusting for the covariates. Model 2 examined survival by immigration, physical activity, and SPD status, adjusting for the covariates. Model 3 assessed survival by the interaction of immigration and SPD status, adjusting for physical activity status and the covariates. Model 4 estimated survival by the interaction of immigration and physical activity status, adjusting for SPD status and the covariates. Model 5 evaluated survival by the interaction of physical activity and SPD status, adjusting for immigration status and the covariates. Model 6 assessed survival by the interaction of immigration, physical activity, and SPD status, adjusting for the covariates. We repeated models 2 and 5, stratified by immigration status. For the statistically significant interaction models, we evaluated the interaction effects by estimating the expected hazard ratios (HRs, also known as relative hazards) or margins using margins in Stata software while adjusting for the covariates. The estimated HRs show the risk of death for each physical activity group across immigration or SPD status. That is, the margins command in Stata is a postestimation command that estimates the expected relative hazard for every combination of physical activity and immigration or SPD status to assess the interaction effects. For example, it can estimate the expected hazard for individuals who engage in sufficient physical activity without SPD. We reported HRs with their 95% CIs with statistical significance set at 2-tailed P<.05. Analyses were weighted, and Taylor series linearization methods were used to account for the stratified, multistage, cluster sampling design of the NHIS.^26^ Analyses were conducted using SUDAAN, version 11.0.4 (RTI International), and Stata, version 18.0 (StataCorp LLC).

## Results

### Characteristics of the Study Population

Among the 587 931 adults included in the analysis, 37.2% (95% CI, 37.0%-37.4%) were aged 35 to 54 years; 51.9% (95% CI, 51.8%-52.1%) were female and 48.1% (95% CI, 47.9%-48.2%) were male; 13.6% (95% CI, 13.3%-14.0%) identified as Hispanic or Latino, 4.4% (95% CI, 4.3%-4.6%) as non-Hispanic Asian, 11.6% (95% CI, 11.3%-11.9%) as non-Hispanic Black, and 69.3% (95% CI, 68.8%-69.7%) as non-Hispanic White; 29.8% (95% CI, 29.6%-30.1%) had completed some college or an associate’s degree; 63.3% (95% CI, 63.1%-63.6%) were employed; 77.1% (95% CI, 76.8%-77.4%) reported being at or above the poverty threshold; 84.8% (95% CI, 84.6%-85.0%) had health insurance coverage; 36.7% (95% CI, 36.2%-37.2%) resided in the Southern US census region; 26.0% (95% CI, 25.8%-26.2%) had obesity; 19.4% (95% CI, 19.2%-19.7%) were current smokers; and 63.3% (95% CI, 63.3%-63.9%) were current alcohol drinkers (Table 1). Most participants identified as nonimmigrant (83.8%; 95% CI, 83.5%-84.1%), engaged in insufficient physical activity (53.7%; 95% CI, 53.4%-54.0%) and had no SPD (96.8%; 95% CI, 96.7%-96.8%) (Table 1). The overall mean follow-up was 10.3 (95% CI, 10.3-10.4) years, which was generally similar across the exposure variables.

**Table 1.** Proportions of the Total Population and Deaths Among US Adults^a^

Characteristic	Total sample at baseline, No. (weighted column %) [95% CI]^a^	Weighted mean follow-up (95% CI), y	Mortality by December 31, 2019, weighted row % (95% CI)
Overall	587 931 (100)	10.3 (10.3-10.4)	11.8 (11.6-11.9)
Exposure variables			
Immigration status			
Immigrant	102 919 (16.2) [15.9-16.5]	10.0 (9.9-10.1)	6.3 (6.1-6.5)
Nonimmigrant	485 012 (83.8) [83.5-84.1]	10.4 (10.3-10.5)	12.8 (12.6-13.0)
Leisure-time physical activity			
Inactive or insufficient	327 799 (53.7) [53.4-54.0]	10.4 (10.4-10.5)	16.1 (15.9-16.3)
Sufficiently active	260 132 (46.3) [46.0-46.6]	10.2 (10.2-10.3)	6.8 (6.6-6.9)
SPD			
Absent	566 743 (96.8) [96.7-96.8]	10.4 (10.3-10.4)	11.6 (11.4-11.7)
Present	21 188 (3.2) [3.2-3.3]	9.4 (9.3-9.6)	18.0 (17.3-18.6)
Covariates			
Age, y			
18-34	165 108 (30.9) [30.6-31.2]	11.2 (11.1-11.3)	1.5 (1.5-1.6)
35-54	209 387 (37.2) [37.0-37.4]	11.2 (11.1-11.3)	5.6 (5.5-5.7)
≥55	213 436 (31.9) [31.6-32.1]	8.5 (8.5-8.6)	28.9 (28.5-29.3)
Sex			
Female	328 182 (51.9) [51.8-52.1]	10.4 (10.3-10.5)	11.2 (11.0-11.4)
Male	259 749 (48.1) [47.9-48.2]	10.3 (10.2-10.3)	12.4 (12.2-12.6)
Self-reported race and ethnicity^b^			
Hispanic or Latino	97 177 (13.6) [13.3-14.0]	9.9 (9.8-10.1)	5.6 (5.3-5.8)
Non-Hispanic African American or Black	82 252 (11.6 [11.3-11.9]	10.2 (10.1-10.4)	11.1 (10.8-11.5)
Non-Hispanic Asian	25 569 (4.4) [4.3-4.6]	9.3 (9.1-9.4)	5.4 (5.0-5.8)
Non-Hispanic White	376 165 (69.3) [68.8-69.7]	10.5 (10.4-10.6)	13.5 (13.3-13.7)
Non-Hispanic other single race and/or multiracial^c^	6351 (1.0) [0.9-1.1]	9.6 (9.1-10.1)	9.8 (8.6-11.2)
Unknown	417 (0.1) [0.1-0.1]	20.1 (19.7-20.5)	14.3 (10.9-18.4)
Educational attainment			
Less than high school	100 449 (14.9) [14.7-15.2]	10.4 (10.3-10.5)	21.2 (20.8-21.6)
High school graduate or GED	158 282 (27.4) [27.1-27.6]	10.6 (10.5-10.7)	14.3 (14.1-14.6)
Technical or some college or associate’s degree	173 195 (29.8 [29.6-30.1]	10.4 (10.3-10.4)	9.1 (8.9-9.3)
College degree or higher	153 425 (27.4) [27.0-27.8]	10.0 (9.9-10.1)	6.8 (6.6-7.0)
Unknown	2580 (0.5) [0.4-0.5]	10.4 (10.1-10.7)	18.3 (16.6-20.1)
Employment status			
Employed	354 126 (63.3) [63.1-63.6]	11.0 (10.9-11.0)	4.9 (4.8-5.0)
Unemployed	233 536 (36.6) [36.4-36.9]	9.2 (9.2-9.3)	23.7 (23.4-24.0)
Unknown	269 (0.1 [0.0-0.1]	12.1 (11.4-12.9)	4.0 (2.2-7.1)
Poverty status			
Below poverty threshold	81 668 (10.5) [10.3-10.7]	9.6 (9.5-9.7)	12.3 (11.9-12.7)
At or above poverty threshold	432 399 (77.1) [76.8-77.4]	10.1 (10.0-10.1)	10.3 (10.1-10.4)
Unknown	73 864 (12.4) [12.2-12.6]	12.7 (12.6-12.8)	20.5 (20.1-20.9)
Insurance coverage			
Insured	495 862 (84.8) [84.6-85.0]	10.2 (10.1-10.2)	12.8 (12.6-13.0)
Uninsured	90 342 (14.8) [14.6-15.0]	11.3 (11.2-11.4)	5.8 (5.7-6.0)
Unknown	1727 (0.4) [0.3-0.4]	9.6 (9.2-10.0)	6.1 (5.1-7.4)
Region of US residence			
Northeast	99 795 (18.0) [17.7-18.4]	10.5 (10.3-10.6)	12.1 (11.7-12.4)
North Central or Midwest	131 905 (23.6) [23.2-24.1]	10.6 (10.5-10.7)	12.1 (11.7-12.4)
South	215 764 (36.7) [36.2-37.2]	10.3 (10.2-10.4)	12.5 (12.2-12.8)
West	140 467 (21.6) [21.2-22.1]	10.0 (9.8-10.1)	9.9 (9.5-10.2)
BMI			
Underweight (<18.5)	10 664 (1.8) [1.8-1.9]	9.9 (9.8-10.1)	19.6 (18.7-20.5)
Normal weight (18.5 to <25.0)	207 109 (35.4) [35.2-35.6]	10.7 (10.7-10.8)	11.5 (11.2-11.7)
Overweight (25.0 to <30)	199 062 (34.0) [33.8-34.1]	10.4 (10.3-10.5)	11.7 (11.5-11.9)
Obesity (≥30.0)	154 293 (26.0) [25.8-26.2]	9.7 (9.6-9.7)	11.8 (11.6-12.0)
Unknown	16 803 (2.8) [2.7-2.9]	10.7 (10.5-10.8)	10.6 (10.1-11.2)
Cigarette smoking status			
Never	338 351 (58.3) [58.1-58.6]	10.2 (10.2-10.3)	8.3 (8.2-8.4)
Former	131 825 (22.0) [21.9-22.2]	9.8 (9.8-9.9)	19.1 (18.8-19.4)
Current	116 712 (19.4) [19.2-19.7]	11.2 (11.1-11.2)	13.8 (13.5-14.1)
Unknown	1043 (0.2) [0.2-0.2]	11.5 (11.0-12.0)	16.7 (14.4-19.3)
Alcohol drinking status			
Lifetime abstinence	129 412 (21.2) [21.0-21.5]	10.4 (10.4-10.5)	14.3 (14.0-14.6)
Former	90 220 (14.3) [14.1-14.4]	9.7 (9.6-9.8)	22.5 (22.1-22.9)
Current	362 979 (63.6) [63.3-63.9]	10.4 (10.4-10.5)	8.5 (8.3-8.6)
Unknown	5320 (0.9) [0.8-0.9]	10.7 (10.5-11.0)	12.7 (11.6-13.8)

Abbreviations: BMI, body mass index (calculated as the weight in kilograms divided by the height in square meters); GED, General Educational Development; SPD, serious psychological distress.

^a^ Data are from the National Health Interview Survey (NHIS) linked to the National Death Index (NDI), 1998 to 2019. Frequencies are unweighted and percentages are weighted.

^b^ The race and ethnicity were categorized using the NHIS NDI linked data.

^c^ Includes American Indian or Alaska Native, Native Hawaiian, or other single and multiple races. These classifications were combined because of the small sample size and to increase statistical power.

### Mortality Rates Between Groups

The overall mortality rate was 11.8% (95% CI, 11.6%-11.9%) by the end of the follow-up. Nonimmigrants, persons who engaged in insufficient physical activity, and those with SPD had higher mortality rates than their corresponding counterparts (Table 1).

### Survival Probabilities by the Intersection of Immigration, Physical Activity, and SPD Status

**Figure 1.**
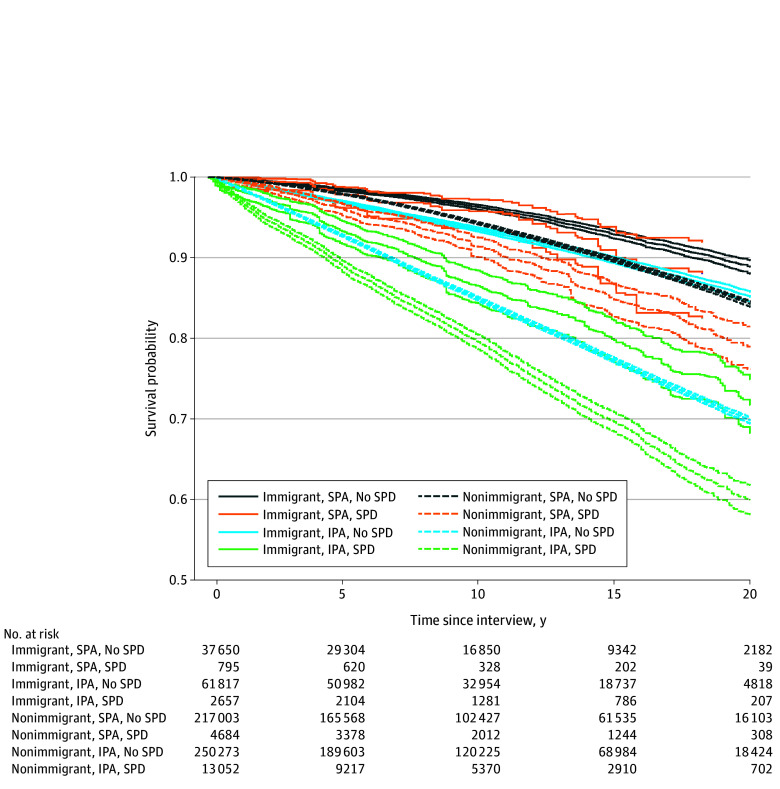
Survival Probabilities by Intersection of Immigration, Physical Activity, and Severe Psychological Distress (SPD) During 22-Year Follow-Up The Kaplan-Meier survival curve estimates survival probabilities from 1998 to 2019 using the intersection of immigration, leisure-time physical activity, and SPD status. IPA indicates insufficient physical activity; SPA, sufficient physical activity. Pointwise 95% CIs are indicated by paler lines of the same pattern and color as the corresponding main line.

Figure 1 shows the Kaplan-Meier curve illustrating survival probabilities during the 22-year follow-up period (NHIS-NDI 1998-2019), estimated by the intersection of immigration, physical activity, and SPD status. Overall, immigrants who engaged in sufficient physical activity and had no SPD had the highest probabilities of surviving during the 22-year follow-up. For instance, their probability of surviving beyond 20 years of the follow-up was 89%. However, nonimmigrants who engaged in insufficient physical activity and had SPD, in general, had the lowest probabilities of survival. These nonimmigrants, for example, had a 60% probability of surviving past 20 years of the follow-up. The corresponding number of people at risk of mortality at different years since the interview is presented in Figure 1.

### Immigration, SPD, and Physical Activity Status Associated With Mortality Risks

The mortality risks associated with immigration, SPD, and physical activity status, without adjusting for the covariates (sociodemographic, socioeconomic, and substance use factors), are presented in Table 2 (model 1). Immigrants had 53% lower mortality risk (HR, 0.47; 95% CI, 0.46-0.49) compared with nonimmigrants. The HR increased to 2.41 (95% CI, 2.36-2.46) in persons who were inactive or insufficiently engaged in leisure-time physical activity (based on the Physical Activity Guidelines for Americans) compared with those who were sufficiently engaged in physical activity. Individuals with SPD had a 54% higher morality risk (HR, 1.46; 95% CI, 1.41-1.52) relative to those without SPD. Even after adjusting for the covariates (eTable 1 in Supplement 1), immigrants still had lower risks (HR, 0.72; 95% CI, 0.69-0.75), while persons with insufficient physical activity (HR, 1.61; 95% CI, 1.58-1.65) and those with SPD (HR, 1.10; 95% CI, 1.05-1.14) also still had higher mortality risk (model 2 in Table 2). However, the HRs were reduced in the adjusted models.

**Table 2.** Cox Proportional Hazard Ratios of Mortality According to Immigration, Physical Activity, and SPD Status^a^

Exposure variable	HR (95% CI)					
	Model 1^b^	Model 2^c^	Model 3^d^	Model 4^e^	Model 5^f^	Model 6^g^
Immigration status						
Immigrant	0.47 (0.46-0.49)^h^	0.72 (0.69-0.75)h	0.72 (0.69-0.75)^h^	0.83 (0.78-0.88)^h^	0.72 (0.69-0.75)^h^	0.84 (0.79-0.89)^h^
Nonimmigrant	1 [Reference]	1 [Reference]	1 [Reference]	1 [Reference]	1 [Reference]	1 [Reference]
Physical activity status						
Inactive or insufficiently active	2.41 (2.36-2.46)^h^	1.61 (1.58-1.65)^h^	1.61 (1.58-1.65)^h^	1.64 (1.60-1.67)^h^	1.62 (1.59-1.66)^h^	1.65 (1.62-1.69)^h^
Sufficiently active	1 [Reference]	1 [Reference]	1 [Reference]	1 [Reference]	1 [Reference]	1 [Reference]
SPD status						
No SPD	1 [Reference]	1 [Reference]	1 [Reference]	1 [Reference]	1 [Reference]	1 [Reference]
SPD	1.46 (1.41-1.52)^h^	1.10 (1.05-1.14)^h^	1.09 (1.05-1.14)^h^	1.09 (1.05-1.14)^h^	1.35 (1.21-1.51)^h^	1.41 (1.25-1.58)^h^

Abbreviations: HR, hazard ratio; SPD, serious psychological distress.

^a^ Data are from the National Health Interview Survey linked to the National Death Index, 1998 to 2019.

^b^ Includes immigration, physical activity, and SPD status.

^c^ Includes immigration, physical activity, and SPD status, adjusting for the covariates (age, sex, race and ethnicity, educational attainment, employment, poverty status, insurance coverage, region of residence, body mass index, cigarette smoking, and alcohol use).

^d^ Includes interaction of immigration status × SPD status (Wald *F*_1_ = 0.11; *P* = .74), adjusting for physical activity and the covariates.

^e^ Includes interaction of immigration status × physical activity status (Wald *F*_1_ = 28.72; *P* < .001), adjusting for SPD and the covariates.

^f^ Includes interaction of physical activity × SPD status (Wald *F*_1_ = 15.88; *P* < .001), adjusting for immigration status and the covariates.

^g^ Includes immigration status × physical activity × SPD status (Wald *F*_4_ = 14.12; *P* < .001), adjusting for the covariates.

^h^ Indicates statistical significance at *P* < .001.

### Intersection of Immigration, Physical Activity, and SPD Status Associated With Mortality Risks

**Figure 2.**
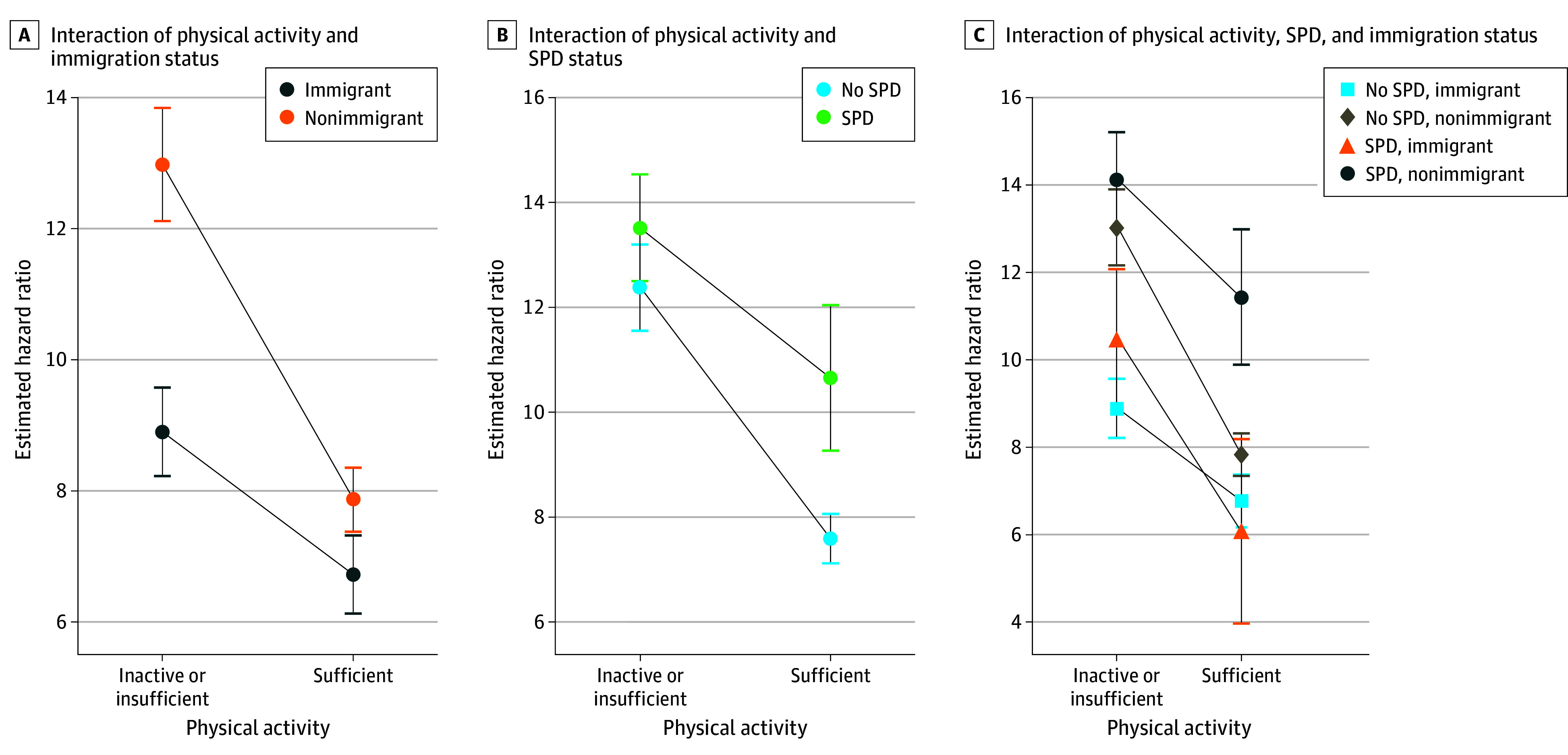
Expected Hazard Ratio of Mortality by Interaction of Physical Activity, Immigration Status, and Serious Psychological Distress (SPD) Status Expected hazard ratios (also known as relative hazards) are adjusted for covariates age, sex, race and ethnicity, educational attainment, employment, poverty status, insurance coverage, region of residence, body mass index, cigarette smoking status, and alcohol drinking status. The expected hazard ratio shows the risk of death for each physical activity group across immigration status with additional adjustment for SPD (A), for each physical activity group across SPD status with additional adjustment for immigration status (B), and for each physical activity group across intersection of SPD and immigration status (C). Margins command, a Stata postestimation command (version 18.0 [StataCorp LLC]), was used to compute the expected relative hazard for every combination of physical activity and immigration (eg, expected hazard for immigrants who engaged in sufficient physical activity [A]), for every combination of physical activity and SPD status (eg, expected hazard for those who engaged in sufficient PA with no SPD [B]), and for each physical activity group across all combinations of immigration and SPD groups (eg, expected hazard for immigrants who engaged in sufficient physical activity with SPD [C]) to assess the interaction effects. The command was implemented after fitting a statistically significant interaction model. Error bars indicate 95% CI.

In model 3 (Table 2) adjusting for physical activity and the covariates, there was no statistically significant interaction between immigration status and SPD (Wald *F*_1_ = 0.11; *P* = .74). In model 4 (Table 2) with adjusting for SPD and the covariates, there was a statistically significant interaction between immigration status and physical activity (Wald *F*_1_ = 28.72; *P* < .001); the expected relative hazards for each physical activity group across immigration status show that immigrants who engaged in sufficient physical activity had the lowest expected mortality risk compared with the expected hazards for nonimmigrants regardless of their physical activity status and immigrants who engaged in insufficient physical activity (Figure 2A). In model 5 (Table 2) with adjusting for immigration and the covariates, there was a statistically significant interaction between physical activity and SPD (Wald *F*_1_ = 15.88; *P* < .001), with the least expected mortality risk among individuals who engaged in sufficient physical activity without SPD compared with the expected hazards for individuals who engaged in insufficient physical activity regardless of SPD status or who engaged in sufficient physical activity with SPD (Figure 2B). There was a statistically significant 3-way interaction among immigration status, physical activity, and SPD status (Wald *F*_4_ = 14.12; *P* < .001), adjusting for the covariates (model 6 in Table 2). The expected relative hazards for each physical activity group across all combinations of immigration and SPD are shown in Figure 2C. There was a lower expected mortality risk among immigrants who engaged in physical activity with SPD (HR, 6.08; 95% CI, 3.97-8.19) compared with immigrants with SPD who lacked physical activity (HR, 10.50; 95% CI, 8.93-12.08) as well as nonimmigrants who lacked physical activity regardless of SPD status (No SPD HR, 13.03; 95% CI, 12.16-13.90 and SPD HR, 14.13; 95% CI, 13.04-15.21) or who engaged in physical activity with SPD (HR, 11.44; 95% CI, 9.89-12.99). Immigrants who engaged in sufficient physical activity with SPD had the lowest expected mortality risk compared with the expected hazards for immigrants who engaged in insufficient physical activity with SPD, or nonimmigrants who engaged in insufficient physical activity regardless of SPD status or who engaged in sufficient physical activity with SPD. However, differences were not significant (due to overlapping 95% CIs) for immigrants who engaged in sufficient physical activity without SPD or who engaged in insufficient physical activity without SPD and nonimmigrants who engaged in sufficient physical activity without SPD (Figure 2C).

### Physical Activity and SPD Status Associated With Mortality Risks by Immigration Status

Insufficient (vs sufficient) physical activity and SPD (vs no SPD) were associated with higher mortality risk among immigrants (model 1 in eTable 2 in Supplement 1) and nonimmigrants (model 3 in eTable 2 in Supplement 1), adjusting for the covariates. The effect size due to insufficient physical activity was higher among nonimmigrants, whereas the effect size due to SPD was higher among immigrants. Among immigrants (model 2 in eTable 2 in Supplement 1), there was no statistically significant interaction between physical activity and SPD (Wald *F*_1_ = 2.58; *P* = .11) with adjusting for the covariates. However, among nonimmigrants (model 4 in eTable 2 in Supplement 1), there was a statistically significant interaction between physical activity and SPD (Wald *F*_1_ = 20.75; *P* < .001) with adjusting for covariates. The expected relative hazards for each physical activity group across SPD status showed that individuals who engaged in sufficient physical activity without SPD had the least expected mortality risk compared with the expected hazards for those who engaged in sufficient physical activity regardless of SPD status or those who engaged in sufficient physical activity with SPD (eFigure 1 in Supplement 1).

## Discussion

This cohort study sought to augment the literature on immigration status, physical activity, SPD, and mortality risks by examining how immigration status and SPD change the direction of the association between physical activity and all-cause mortality risks using a nationally representative sample of US adults. We found an association with mortality risk among immigration, physical activity, and SPD status. Notably, nonimmigrants, persons who engaged in insufficient physical activity, and those with SPD had higher mortality risks. The findings also showed no significant interaction between immigration status and SPD. However, significant interactions were observed between immigration status and physical activity, physical activity and SPD, and their combined association, indicating unique mortality risks influenced by the synergy of these factors. There appears to be a mitigating effect of immigration status and physical activity among individuals without SPD. Specifically, immigrants who engaged in sufficient physical activity without SPD had the highest probability of survival beyond the 22-year follow-up.

The mitigating effect of immigration status in this study may reflect protective characteristics associated with immigrant populations, supporting the healthy immigrant effect or advantage. It is well-known that immigrants generally have better health outcomes than domestic-born individuals in their destination countries,^12,13,33,34,35,36,37^ due to positive selection of healthier immigrants into destination countries and immigrants’ resilience.^33,34,35,36,37^ Immigrants are often self-selected persons or those selected (through strict migration processes) with better health and social outcomes, giving them a health advantage over nonimmigrants.^33,34,35,36,37^ Immigrants, especially newcomers who are positively chosen, have strong resilience that is reflected in their motivation and determination to succeed in their destination countries by effectively seeking and using key resources and community assets that enable them to maintain their health advantage.^34,37^ The health advantage and lower mortality risks are also likely due to out-migration of unhealthier immigrants, known as the salmon bias hypothesis.^38,39^ Existing evidence suggests that immigrants with deteriorating or poorer health often return to their countries of origin, resulting in lower number of unhealthy immigrants and reduced risks of mortality in the destination countries.^38,39^ The salmon bias or unhealthy out-migration hypothesis may also explain the health advantage of immigrants, though they experience higher levels of socioeconomic disadvantages (eg, lack of health care access, unemployment, language barrier, discrimination) in their destination countries.^12,13,38,39^

The synergistic effects of immigration, physical activity, and SPD status in the risks of mortality provide some understanding of the health advantage of US immigrants compared with their US-born counterparts. Thus, immigration status may be a critical social determinant of health, as immigration status changed and attenuated the effects of physical activity and SPD on mortality in this study. It is possible that immigrant health advantages outweigh other health benefits. While lack of physical activity and SPD were associated with higher mortality risk, which is consistent with previous studies,^1,2,3,8,9^ their intersection with immigration status attenuated their negative effects. For instance, immigrants with SPD who engaged in sufficient physical activity had lower mortality risks relative to their immigrant counterparts with SPD who engaged in insufficient physical activity, as well as nonimmigrants with similar physical activity and SPD profiles. However, their lower risks were not significantly different from those of immigrants without SPD who engaged in sufficient physical activity or those without SPD who engaged in insufficient physical activity and those of nonimmigrants without SPD who engaged in sufficient physical activity. When stratified by immigration status, lack of physical activity and SPD remained significant risk factors for mortality among both immigrants and nonimmigrants. However, while the interaction between physical activity and SPD was not significant among immigrant population, it was significant among the nonimmigrant population, emphasizing the heterogeneity and health disparities in population subgroups. That is, among nonimmigrants, individuals who engaged in sufficient physical activity with or without SPD or those who engaged in sufficient physical activity with SPD exhibited higher mortality risks than those who engaged in sufficient physical activity without SPD. In contrast, these differences were not observed among immigrants. These findings indicate the utility of disaggregating data to examine and delineate disparities in mortality risk to improve tailored risk-reduction intervention efforts.

Future considerations of our findings may include tailored lifestyle physical activity promotion interventions delivered via web, text, and community-based platforms that are accessible to all immigrant and nonimmigrant communities.^40,41,42,43^ Lifestyle physical activity interventions (eg, brief physical activity interventions), whether delivered in health care or non–health care settings, have been shown to effectively increase self-reported and objectively measured physical activity levels.^44,45,46^ Additionally, increased physical activity serves as both a protective factor and an intervention for mental health issues, further enhancing the direct and indirect benefits of brief physical activity–based interventions.^47^

### Limitations

Our study has some limitations. All measures (except for mortality status) were self-reported, which could have introduced self-report bias (eg, social desirability, acquiescence). Second, data were not available to estimate time-varying covariates and coefficients to determine the changing effects of the exposures (eg, physical activity, SPD, substance use). Third, although we included several factors or covariates in our analyses, residual confounders (eg, antipsychotic medication use, health status or medical conditions at baseline, and health care access) may have altered the observed associations. Fourth, we assessed leisure-time physical activity, which may not fully capture total physical activity levels, especially among immigrants who often work in physically demanding industries or occupations.^48,49^ However, work-related physical activity was not captured in this study. Fifth, the NHIS did not collect information on immigrants’ legal status or subgroups (eg, undocumented immigrants), and therefore we could not estimate detailed mortality risks within these categories. To protect participant confidentiality, the NHIS public-use data also did not include information on respondents’ specific country of birth or origin. Sixth, our analysis did not explore time since migration, a factor shown to affect the interaction between immigration status and health outcomes.^50,51,52,53^ Finally, the Kessler Psychological Distress Scale is not a diagnostic tool and therefore is not equivalent to a clinical diagnosis.

## Conclusions

In this cohort study of a nationally representative sample of immigrant and nonimmigrant adult populations in the US, the results showed a joint association of immigration, physical activity, and SPD status with mortality risks. The findings suggest the need to consider immigration status in behavioral health interventions (eg, exercise, psychoeducation) aimed at reducing mortality risks among individuals who lack physical activity and/or experience SPD, thereby mitigating the mortality gap or disparities in the population.

## Notes

Supplement 1. **eTable 1.** Cox Hazard Ratios of Mortality According to Sociodemographic, Mental Health, and Behavioral Factors, NHIS-NDI 1998 to 2019 (N = 587 931) **eTable 2.** Cox Hazard Ratios of Mortality Among Immigrant and Nonimmigrant Adults, NHIS-NDI 1998 to 2019 **eFigure.** Predicted Hazard Ratio of Mortality by Interaction of Physical Activity and SPD Among Nonimmigrants

Supplement 2.
Data Sharing Statement
