# Supplementary material for: Interaction Between Immigration, Physical Activity, Mental Health, and All-Cause Mortality Among US Adults
Source: JAMA Netw Open. 2025 Oct 8;8(10):e2536371. doi: 10.1001/jamanetworkopen.2025.36371 (PMC12509011; doi:10.1001/jamanetworkopen.2025.36371)
Supplement: Supplement 1. — eTable 1. Cox Hazard Ratios of Mortality According to Sociodemographic, Mental Health, and Behavioral Factors, NHIS-NDI 1998 to 2019 (N = 587 931) eTable 2. Cox Hazard Ratios of Mortality Among Immigrant and Nonimmigrant Adults, NHIS-NDI 1998 to 2019 eFigure. Predicted Hazard Ratio of Mortality by Interaction of Physical Activity and SPD Among Nonimmigrants [file jamanetwopen-e2536371-s001.pdf]

Supplementary Online Content

Adzrago D, McNeel TS, Dyer TV, et al. Interaction of immigration, physical activity, mental health, and all-cause mortality among US adults. *JAMA Netw Open.* 2025;8(10):e2536371. doi:10.1001/jamanetworkopen.2025.36371

eTable 1. Cox Hazard Ratios of Mortality According to Sociodemographic, Mental Health, and Behavioral Factors, NHIS-NDI 1998 to 2019 (N = 587 931)

eTable 2. Cox Hazard Ratios of Mortality Among Immigrant and Nonimmigrant Adults, NHIS-NDI 1998 to 2019

eFigure. Predicted Hazard Ratio of Mortality by Interaction of Physical Activity and SPD Among Nonimmigrants

This supplementary material has been provided by the authors to give readers additional information about their work.

© 2025 Adzrago D et al. *JAMA Network Open*.

eTable 1. Cox Hazard Ratios of Mortality According to Sociodemographic, Mental Health, and Behavioral Factors, NHIS-NDI 1998 to 2019 (N = 587 931)

	Model 1[a]	Model 2[b]	Model 3[c]	Model 4[d]	Model 5[e]	Model 6[f]
	**HR (95% CI)**	**HR (95% CI)**	**HR (95% CI)**	**HR (95% CI)**	**HR (95% CI)**	**HR (95% CI)**
**Exposure variables**						
**Immigration**						
Immigrant	0.47 (0.46-0.49)[g]	0.72 (0.69-0.75)[g]	0.72 (0.69-0.75)[g]	0.83 (0.78-0.88)[g]	0.72 (0.69-0.75)[g]	0.84 (0.79-0.89)[g]
Non-immigrant	1 [Reference]	1 [Reference]	1 [Reference]	1 [Reference]	1 [Reference]	1 [Reference]
**PA**						
Inactive/insufficient	2.41 (2.36-2.46)[g]	1.61 (1.58-1.65)[g]	1.61 (1.58-1.65)[g]	1.64 (1.60-1.67)[g]	1.62 (1.59-1.66)[g]	1.65 (1.62-1.69)[g]
Sufficiently active	1 [Reference]	1 [Reference]	1 [Reference]	1 [Reference]	1 [Reference]	1 [Reference]
**SPD**						
No SPD	1 [Reference]	1 [Reference]	1 [Reference]	1 [Reference]	1 [Reference]	1 [Reference]
SPD	1.46 (1.41-1.52)[g]	1.10 (1.05-1.14)[g]	1.09 (1.05-1.14)[g]	1.09 (1.05-1.14)[g]	1.35 (1.21-1.51)[g]	1.41 (1.25-1.58)[g]
**Immigration X SPD**	NA	NA	Wald F (1) = 0.11, *P*= 0.735	NA	NA	NA
**Immigration X PA**	NA	NA	NA	Wald F (1) = 28.72, *P*<.001	NA	NA
**PA X SPD**	NA	NA	NA	NA	Wald F (1) = 15.88, *P*<.001	NA
**Immigration X PA X SPD**	NA	NA	NA	NA	NA	Wald F (4) = 14.12, *P*<.001
**Covariates**						
**Age**	NA					
18 - 34 years	NA	1 [Reference]	1 [Reference]	1 [Reference]	1 [Reference]	1 [Reference]
35 - 54 years	NA	3.87 (3.66-4.09)[g]	3.87 (3.66-4.09)[g]	3.87 (3.66-4.09)[g]	3.87 (3.66-4.09)[g]	3.87 (3.66-4.09)[g]
55 years or older	NA	14.92 (14.17-15.71)[g]	14.92 (14.16-15.71)[g]	14.92 (14.17-15.71)[g]	14.93 (14.18-15.73)[g]	14.94 (14.19-15.73)[g]
**Sex**	NA					
Female	NA	0.67 (0.66-0.69)[g]	0.67 (0.66-0.69)[g]	0.67 (0.66-0.69)[g]	0.67 (0.66-0.69)[g]	0.67 (0.66-0.68)[g]
Male	NA	1 [Reference]	1 [Reference]	1 [Reference]	1 [Reference]	1 [Reference]
**Race and/or ethnicity**[j]	NA					

© 2025 Adzrago D et al. *JAMA Network Open*.

	Model 1[a]	Model 2[b]	Model 3[c]	Model 4[d]	Model 5[e]	Model 6[f]
	HR (95% CI)	HR (95% CI)	HR (95% CI)	HR (95% CI)	HR (95% CI)	HR (95% CI)
Non-Hispanic African American or Black	NA	0.91 (0.89-0.94)[g]	0.91 (0.89-0.94)[g]	0.91 (0.89-0.94)[g]	0.91 (0.89-0.94)[g]	0.91 (0.89-0.94)[g]
Non-Hispanic Asian	NA	0.71 (0.67-0.76)[g]	0.71 (0.67-0.76)[g]	0.71 (0.66-0.76)[g]	0.71 (0.67-0.76)[g]	0.71 (0.66-0.76)[g]
Hispanic or Latino	NA	0.68 (0.65-0.70)[g]	0.68 (0.65-0.70)[g]	0.68 (0.65-0.71)[g]	0.68 (0.65-0.70)[g]	0.68 (0.65-0.71)[g]
Non-Hispanic Other Single and/or Multi race	NA	0.88 (0.78-0.99)[h]	0.88 (0.78-0.99)[h]	0.88 (0.78-0.98)[h]	0.88 (0.78-0.99)[h]	0.88 (0.78-0.98)[h]
Non-Hispanic White	NA	1 [Reference]	1 [Reference]	1 [Reference]	1 [Reference]	1 [Reference]
Unknown	NA	1.18 (0.88-1.58)	1.18 (0.88-1.58)	1.20 (0.90-1.61)	1.18 (0.88-1.58)	1.20 (0.90-1.61)
**Education**	NA					
Less than High School	NA	1 [Reference]	1 [Reference]	1 [Reference]	1 [Reference]	1 [Reference]
High School graduate/GED	NA	0.78 (0.76-0.79)[g]	0.78 (0.76-0.79)[g]	0.78 (0.76-0.79)[g]	0.78 (0.76-0.79)[g]	0.78 (0.76-0.79)[g]
Technical or Some college/AA degree	NA	0.70 (0.68-0.72)[g]	0.70 (0.68-0.72)[g]	0.70 (0.68-0.72)[g]	0.70 (0.68-0.72)[g]	0.70 (0.68-0.72)[g]
College degree or higher	NA	0.57 (0.55-0.59)[g]	0.57 (0.55-0.59)[g]	0.57 (0.55-0.59)[g]	0.57 (0.55-0.59)[g]	0.57 (0.55-0.59)[g]
Unknown	NA	0.87 (0.78-0.97)[h]	0.87 (0.78-0.97)[h]	0.87 (0.78-0.97)[h]	0.87 (0.78-0.97)[h]	0.87 (0.78-0.97)[h]
**Employment**	NA					
Employed	NA	1 [Reference]	1 [Reference]	1 [Reference)	1 [Reference]	1 [Reference]
Unemployed	NA	2.64 (2.58-2.70)[g]	2.64 (2.58-2.70)[g]	2.64 (2.58-2.70)[g]	2.64 (2.58-2.70)[g]	2.64 (2.58-2.70)[g]
Unknown	NA	0.54 (0.29-1.00)	0.54 (0.29-1.00)	0.54 (0.29-1.00)	0.54 (0.29-1.00)	0.54 (0.29-1.00)
**Poverty status**	NA					
Below poverty threshold	NA	1.043 (1.01-1.07)[i]	1.04 (1.01-1.07)[h]	1.04 (1.01-1.08)[i]	1.043 (1.01-1.07)[i]	1.05 (1.02-1.08)[i]
At or above poverty threshold	NA	1 [Reference]	1 [Reference]	1 [Reference]	1 [Reference]	1 [Reference]
**Unknown**	NA	1.18 (1.15-1.20)[g]	1.18 (1.15-1.20)[g]	1.18 (1.15-1.20)[g]	1.18 (1.15-1.20)[g]	1.18 (1.15-1.20)[g]
**Insurance coverage**	NA					
Insured	NA	1 [Reference]	1 [Reference]	1 [Reference]	1 [Reference]	1 [Reference]
Uninsured	NA	0.75 (0.73-0.78)[g]	0.75 (0.73-0.78)[g]	0.75 (0.73-0.78)[g]	0.75 (0.73-0.78)[g]	0.75 (0.73-0.78)[g]

© 2025 Adzrago D et al. *JAMA Network Open*.

	Model 1[a] HR (95% CI)	Model 2[b] HR (95% CI)	Model 3[c] HR (95% CI)	Model 4[d] HR (95% CI)	Model 5[e] HR (95% CI)	Model 6[f] HR (95% CI)
Unknown	NA	0.77 (0.64-0.94)[i]	0.77 (0.64-0.94)[i]	0.77 (0.64-0.94)[i]	0.77 (0.64-0.94)[i]	0.77 (0.64-0.94)[i]
**Region of residence**	NA					
Northeast	NA	1.00 (0.97-1.03)	1.00 (0.97-1.03)	1.00 (0.98-1.03)	1.00 (0.97-1.03)	1.01 (0.98-1.03)
North Central/Midwest	NA	0.99 (0.97-1.02)	0.99 (0.97-1.02)	0.99 (0.97-1.02)	0.99 (0.97-1.02)	0.99 (0.97-1.02)
South	NA	1 [Reference]	1 [Reference]	1 [Reference]	1 [Reference]	1 [Reference]
West	NA	1.01 (0.98-1.04)	1.01 (0.98-1.04)	1.01 (0.98-1.04)	1.01 (0.98-1.04)	1.01 (0.98-1.04)
**Body mass index (BMI)**	NA					
Underweight (BMI < 18.5)	NA	1.83 (1.73-1.93)[g]	1.83 (1.73-1.93)[g]	1.83 (1.73-1.92)[g]	1.83 (1.73-1.93)[g]	1.83 (1.73-1.93)[g]
Normal weight (BMI >= 18.5 & BMI < 25)	NA	1 [Reference]	1 [Reference]	1 [Reference]	1 [Reference]	1 [Reference]
Overweight (BMI >= 25 & BMI < 30)	NA	0.81 (0.80-0.83)[g]	0.81 (0.80-0.83)[g]	0.81 (0.80-0.83)[g]	0.81 (0.79-0.83)[g]	0.81 (0.79-0.83)[g]
Obese (BMI 30+)	NA	0.80 (0.78-0.82)[g]	0.80 (0.78-0.82)[g]	0.80 (0.78-0.82)[g]	0.80 (0.78-0.82)[g]	0.80 (0.78-0.82)[g]
Unknown	NA	0.73 (0.69-0.76)[g]	0.73 (0.69-0.76)[g]	0.73 (0.69-0.77)[g]	0.73 (0.69-0.76)[g]	0.73 (0.69-0.77)[g]
**Cigarette smoking status**	NA					
Never smoked	NA	1 [Reference]	1 [Reference]	1 [Reference]	1 [Reference]	1 [Reference]
Former smoker	NA	1.34 (1.31-1.36)[g]	1.34 (1.31-1.36)[g]	1.34 (1.31-1.36)[g]	1.34 (1.31-1.36)[g]	1.34 (1.31-1.36)[g]
Current smoker	NA	1.60 (1.56-1.64)[g]	1.60 (1.56-1.64)[g]	1.60 (1.56-1.64)[g]	1.60 (1.56-1.64)[g]	1.60 (1.56-1.64)[g]
Unknown	NA	1.36 (1.15-1.61)[g]	1.36 (1.15-1.61)[g]	1.36 (1.15-1.60)[g]	1.36 (1.15-1.60)[g]	1.36 (1.15-1.60)[g]
**Alcohol drinking status**	NA					
Lifetime abstainer	NA	1 [Reference]	1 [Reference]	1 [Reference]	1 [Reference]	1 [Reference]
Former drinker	NA	1.00 (0.98-1.03)	1.00 (0.98-1.03)	1.00 (0.98-1.03)	1.00 (0.98-1.03)	1.00 (0.98-1.03)
Current drinker	NA	0.74 (0.72-0.76)[g]	0.74 (0.72-0.76)[g]	0.74 (0.72-0.76)[g]	0.74 (0.72-0.76)[g]	0.74 (0.72-0.76)[g]
Unknown	NA	0.76 (0.69-0.83)[g]	0.76 (0.69-0.83)[g]	0.76 (0.69-0.83)[g]	0.76 (0.69-0.83)[g]	0.76 (0.69-0.83)[g]

© 2025 Adzrago D et al. *JAMA Network Open*.

Abbreviations: NHIS, National Health Interview Survey; NDI, National Death Index; PA, leisure-time physical activity; SPD, serious psychological distress; HR, hazard ratio; 95% CI, 95% confidence interval; NA, not applicable.

[a] Model 1= Immigration status + PA + SPD.

[b] Model 2= Immigration status + PA + SPD, adjusting for the covariates (age, sex, race and/or ethnicity, education, employment, poverty status, insurance coverage, region of residence, BMI, cigarette smoking, and alcohol use).

[c] Model 3= Immigration status X SPD, adjusting for PA and the covariates.

[d] Model 4= Immigration status X PA, adjusting for SPD and the covariates.

[e] Model 5= PA X SPD, adjusting for immigration status and the covariates.

[f] Model 6= Immigration status X PA X SPD, adjusting for the covariates.

[g] Indicates statistical significance at $P < .001$.

[h] Indicates statistical significance at $P < .05$.

[i] Indicates statistical significance at $P < .01$.

[j] Race and/or ethnicity were self-reported and classified as non-Hispanic African American or Black, non-Hispanic Asian, Hispanic or Latino, non-Hispanic Other Single and/or Multiple race (Alaska Native or Native American and Native Hawaiian, or other single and multiple races), and non-Hispanic White.

© 2025 Adzrago D et al. *JAMA Network Open*.

eTable 2. Cox Hazard Ratios of Mortality Among Immigrant and Non-immigrant Adults, NHIS-NDI 1998 to 2019

	**Immigrant** (n= 102,919)		**Non-immigrant** (n= 485,012)	
	**Model 1[a]**	**Model 2[b]**	**Model 3[c]**	**Model 4[d]**
	**HR (95% CI)**	**HR (95% CI)**	**HR (95% CI)**	**HR (95% CI)**
**Exposure variables**				
**PA**				
Inactive/insufficient	1.42 (1.32-1.52)[e]	1.40 (1.31-1.51)[e]	1.63 (1.60-1.67)[e]	1.65 (1.61-1.68)[e]
Sufficiently active	1 [Reference]	1 [Reference]	1 [Reference]	1 [Reference]
**SPD**				
No SPD	1 [Reference]	1 [Reference]	1 [Reference]	1 [Reference]
SPD	1.18 (1.05-1.34)[f]	0.92 (0.66-1.28)	1.08 (1.04-1.13)[e]	1.40 (1.24-1.57)[e]
**PA X SPD**	NA	Wald F (1) = 2.58, *P*=.107	NA	Wald F (1) = 20.75, *P*<.001
**Covariates**				
**Age**				
18 - 34 years	1 [Reference]	1 (Reference]	1 [Reference]	1 [Reference]
35 - 54 years	2.80 (2.45-3.20)[e]	2.80 (2.45-3.20)[e]	4.06 (3.82-4.30)[e]	4.06 (3.83-4.31)[e]
55 years or older	11.92 (10.55-13.47)[e]	11.92 (10.55-13.47)[e]	15.44 (14.61-16.32)[e]	15.46 (14.63-16.35)[e]
**Sex**				
Female	0.65 (0.62-0.69)[e]	0.65 (0.62-0.69)[e]	0.68 (0.66-0.69)[e]	0.67 (0.66-0.69)[e]
Male	1 [Reference]	1 [Reference]	1 [Reference]	1 [Reference]
**Race and/or ethnicity [h]**				
Non-Hispanic African American or Black	0.64 (0.56-0.73)[e]	0.64 (0.56-0.73)[e]	0.93 (0.90-0.96)[e]	0.93 (0.90-0.95)[e]
Non-Hispanic Asian	0.67 (0.61-0.73)[e]	0.67 (0.61-0.73)[e]	0.75 (0.67-0.84)[e]	0.75 (0.67-0.84)[e]
Hispanic or Latino	0.60 (0.55-0.65)[e]	0.60 (0.55-0.65)[e]	0.73 (0.69-0.76)[e]	0.73 (0.69-0.76)[e]
Non-Hispanic Other Single and/or Multi race	0.66 (0.43-1.02)	0.66 (0.43-1.02)	0.89 (0.79-1.00)	0.89 (0.79-1.00)
Non-Hispanic White	1 [Reference]	1 [Reference]	1 [Reference]	1 [Reference]
Unknown	0.95 (0.65-1.39)	0.95 (0.65-1.39)	1.56 (1.03-2.37)[g]	1.57 (1.03-2.37)[g]
**Education**				
Less than High School	1 [Reference]	1 [Reference]	1 [Reference]	1 [Reference]
High School graduate/GED	0.83 (0.77-0.90)[e]	0.83 (0.77-0.90)[e]	0.77 (0.75-0.79)[e]	0.77 (0.75-0.79)[e]
Technical or Some college/AA degree	0.81 (0.74-0.88)[e]	0.81 (0.74-0.88)[e]	0.69 0.67-0.71)[e]	0.69 (0.67-0.71)[e]
College degree or higher	0.59 (0.54-0.65)[e]	0.59 (0.53-0.65)[e]	0.56 (0.55-0.58)[e]	0.57 (0.55-0.58)[e]
Unknown	1.08 (0.86-1.35)	1.08 (0.86-1.35)	0.83 (0.73-0.94)[f]	0.82 (0.72-0.94)[f]
**Employment**				
Employed	1 [Reference]	1 [Reference]	1 [Reference]	1 [Reference]

© 2025 Adzrago D et al. *JAMA Network Open*.

	**Immigrant** (n= 102,919)		**Non-immigrant** (n= 485,012)	
	**Model 1[a]**	**Model 2[b]**	**Model 3[c]**	**Model 4[d]**
	**HR (95% CI)**	**HR (95% CI)**	**HR (95% CI)**	**HR (95% CI)**
Unemployed	2.46 (2.30-2.63)[e]	2.46 (2.30-2.63)[e]	2.65 (2.59-2.72)[e]	2.66 (2.59-2.72)[e]
Unknown	<0.01 (<0.01-0.01)[e]	<0.01 (<0.01-0.01)[e]	0.61 (0.33-1.15)	0.61 (0.33-1.15)
**Poverty status**				
Below poverty threshold	1.03 (0.95-1.11)	1.03 (0.95-1.11)	1.05 (1.02-1.08)[f]	1.05 (1.02-1.08)[f]
At or above poverty threshold	1 [Reference]	1 [Reference]	1 [Reference]	1 [Reference]
Unknown	1.19 (1.11-1.28)[e]	1.19 (1.11-1.28)[e]	1.17 (1.15-1.20)[e]	1.17 (1.15-1.20)[e]
**Insurance coverage**				
Insured	1 [Reference]	1 (Reference]	1 [Reference]	1 [Reference]
Uninsured	0.65 (0.59-0.71)[e]	0.65 (0.59-0.71)[e]	0.78 (0.75-0.81)[e]	0.78 (0.75-0.81)[e]
Unknown	1.18 (0.72-1.94)	1.18 (0.72-1.94)	0.72 (0.58-0.89)[f]	0.72 (0.58-0.89)[f]
**Region of residence**				
Northeast	0.86 (0.79-0.93)[e]	0.86 (0.79-0.93)[e]	1.03 (1.00-1.06)	1.03 (1.00-1.06)[g]
North Central/Midwest	0.89 (0.81-0.99)[g]	0.89 (0.81-0.99)[g]	1.00 (0.98-1.03)	1.00 (0.98-1.03)
South	1 [Reference]	1 [Reference]	1 [Reference]	1 [Reference]
West	0.97 (0.90-1.04)	0.97 (0.90-1.04)	1.01 (0.98-1.04)	1.01 (0.98-1.04)
**Body mass index (BMI)**				
Underweight (BMI < 18.5)	1.68 (1.40-2.03)[e]	1.69 (1.40-2.03)[e]	1.84 (1.74-1.94)[e]	1.84 (1.74-1.94)[e]
Normal weight (BMI >= 18.5 & BMI < 25)	1 [Reference]	1 [Reference]	1 [Reference]	1 [Reference]
Overweight (BMI >= 25 & BMI < 30)	0.86 (0.81-0.92)[e]	0.86 (0.81-0.92)[e]	0.81 (0.79-0.83)[e]	0.81 (0.79-0.83)[e]
Obese (BMI 30+)	0.91 (0.83-0.98)[g]	0.91 (0.84-0.98)[g]	0.79 (0.77-0.81)[e]	0.79 (0.77-0.81)[e]
Unknown	0.88 (0.75-1.03)	0.88 (0.75-1.03)	0.71 (0.67-0.75)[e]	0.71 (0.67-0.75)[e]
**Cigarette smoking status**				
Never smoked	1 [Reference]	1 [Reference]	1 [Reference]	1 [Reference]
Former smoker	1.34 (1.26-1.44)[e]	1.34 (1.26-1.44)[e]	1.33 (1.31-1.36)[e]	1.33 (1.31-1.36)[e]
Current smoker	1.53 (1.42-1.66)[e]	1.54 (1.42-1.66)[e]	1.59 (1.55-1.63)[e]	1.59 (1.55-1.63)[e]
Unknown	2.16 (1.30-3.58)[f]	2.18 (1.32-3.61)[f]	1.30 (1.09-1.55)[f]	1.30 (1.09-1.55)[f]
**Alcohol drinking status**				
Lifetime abstainer	1 [Reference]	1 [Reference]	1 [Reference]	1 [Reference]
Former drinker	1.21 (1.12-1.31)[e]	1.21 (1.12-1.31)[e]	0.98 (0.95-1.00)	0.98 (0.95-1.00)
Current drinker	0.84 (0.78-0.90)[e]	0.84 (0.78-0.90)[e]	0.73 (0.71-0.74)[e]	0.73 (0.71-0.74)[e]
Unknown	0.84 (0.62-1.14)	0.84 (0.62-1.14)	0.74 (0.67-0.83)[e]	0.75 (0.67-0.83)[e]

Abbreviations: NHIS, National Health Interview Survey; NDI, National Death Index; PA, leisure-time physical activity; SPD, serious psychological distress; HR, hazard ratio; 95% CI, 95% confidence interval; NA, not applicable.

[a] Model 1= PA + SPD, adjusting for the covariates (age, sex, race and/or ethnicity, education, employment, poverty status, insurance coverage, region of residence, BMI, cigarette smoking, and alcohol use) among immigrants.

[b] Model 2= PA X SPD, adjusting for the covariates among immigrants.

© 2025 Adzrago D et al. *JAMA Network Open*.

[c] Model 3= PA + SPD, adjusting for the covariates among non-immigrants.

[d] Model 4= PA X SPD, adjusting for the covariates among non-immigrants.

[e] Indicates statistical significance at *P* < .001.

[f] Indicates statistical significance at *P* < .01.

[g] Indicates statistical significance at *P* < .05.

[h] Race and/or ethnicity were self-reported and classified as non-Hispanic African American or Black, non-Hispanic Asian, Hispanic or Latino, non-Hispanic Other Single and/or Multiple race (Alaska Native or Native American and Native Hawaiian, or other single and multiple races), and non-Hispanic White.

© 2025 Adzrago D et al. *JAMA Network Open*.

eFigure. Predicted Hazard Ratio of Mortality by Interaction of Physical Activity and SPD Among Nonimmigrants

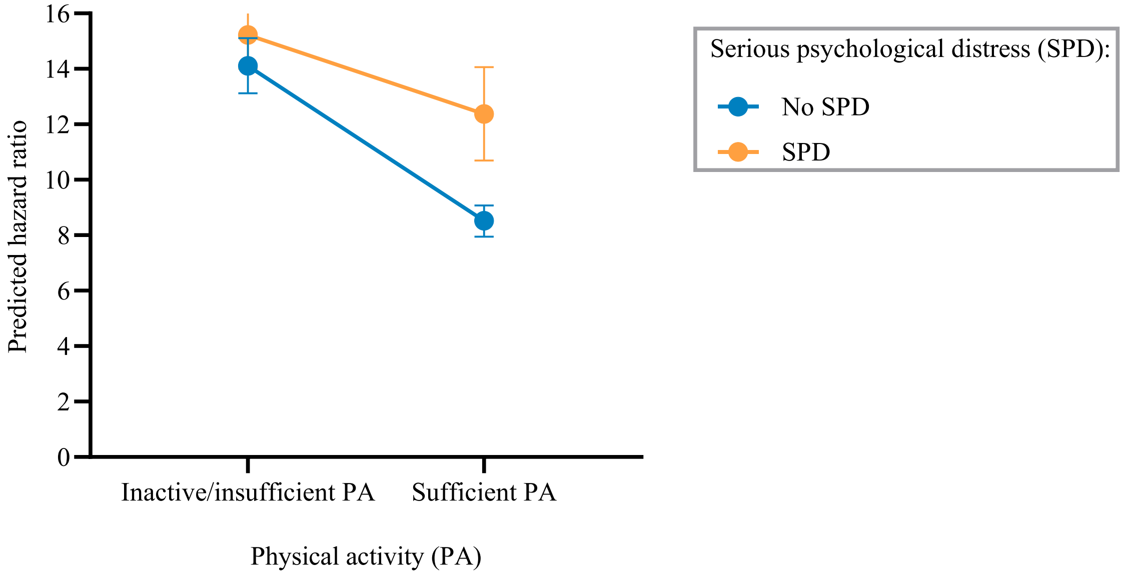

Predicted hazard ratios (also known as relative hazards) are adjusted for age, sex, race and/or ethnicity, education, employment, poverty status, insurance coverage, region of residence, body mass index, cigarette smoking status, and alcohol drinking status. The predicted hazard ratio shows the risk of death for each PA group across SPD status. That is, margins command, a Stata postestimation command, was used to compute the expected relative hazard for every combination of PA and SPD groups (e.g., expected hazard for those who engaged in sufficient PA with SPD) to assess the interaction effects. The command was implemented after fitting a statistically significant interaction model.

© 2025 Adzrago D et al. *JAMA Network Open*.
