# Supplementary material for: Interaction Between Immigration, Physical Activity, Mental Health, and All-Cause Mortality Among US Adults
Source: JAMA Netw Open. 2025 Oct 8;8(10):e2536371. doi: 10.1001/jamanetworkopen.2025.36371 (PMC12509011; doi:10.1001/jamanetworkopen.2025.36371)
Supplement: Supplement 2. — Data Sharing Statement [file jamanetwopen-e2536371-s002.pdf]

Data Sharing Statement

Adzrago. Interaction Between Immigration, Physical Activity, Mental Health, and All-Cause Mortality Among US Adults. *JAMA Netw Open*. Published October 08, 2025. doi:10.1001/jamanetworkopen.2025.36371

Data
Data available: Yes
Data types: Deidentified participant data
How to access data: The datasets analyzed are publicly available in the CDC database repository, https://www.cdc.gov/nchs/nhis/data-questionnaires-documentation.htm
When available: With publication

Supporting Documents
Document types: Other (please specify)
Additional Information: The datasets analyzed are publicly available in the CDC database repository, https://www.cdc.gov/nchs/nhis/data-questionnaires-documentation.htm
How to access documents: The datasets analyzed are publicly available in the CDC database repository, https://www.cdc.gov/nchs/nhis/data-questionnaires-documentation.htm
When available: With publication

Additional Information
Who can access the data: The datasets analyzed are publicly available to anyone in the CDC database repository, https://www.cdc.gov/nchs/nhis/data-questionnaires-documentation.htm
Types of analyses: The datasets analyzed are publicly available to any researcher in the CDC database repository, https://www.cdc.gov/nchs/nhis/data-questionnaires-documentation.htm
Mechanisms of data availability: The datasets analyzed are publicly available in the CDC database repository, https://www.cdc.gov/nchs/nhis/data-questionnaires-documentation.htm
